# Statement of Retraction: Circular RNA CircSHKBP1 accelerates the proliferation, invasion, angiogenesis, and stem cell-like properties via modulation of microR-766-5p/high mobility group AT-hook 2 axis in laryngeal squamous cell carcinoma

**DOI:** 10.1080/21655979.2026.2729939

**Published:** 2026-10-01

We, the Editors and Publisher of the journal *Bioengineered*, have retracted the following article from publication.

Chen, F., Zhang, H., & Wang, J. (2022). Circular RNA CircSHKBP1 accelerates the proliferation, invasion, angiogenesis, and stem cell-like properties via modulation of microR-766-5p/high mobility group AT-hook 2 axis in laryngeal squamous cell carcinoma. *Bioengineered*, 13(5), 11551–11563. https://doi.org/10.1080/21655979.2022.2068922

Since publication, significant concerns have been raised by a third party about the integrity of the images in the article, including concerns about alleged image duplication with similar images from unrelated articles:

- Figure 5d TU686/si-circSHKBP1 panel appears to be duplicated with part of a similar image in Figure 3B SCC 12/50 µM panel from Wang et al, 2020 (https://doi.org/10.2147/OTT.S251806), and with part of a similar image in Figure 3B miR-377 mimic panel from Wang et al, 2021 (https://doi.org/10.21037/atm-20-8027).
- Figure 5d TU686/si-circSHKBP1 + miR-766-5p inhibitor panel appears to be duplicated with part of a similar image in Figure 3 Wogonoside 0 µM panel from Yan et al, 2020 (https://doi.org/10.21037/jtd-20-1555).
- Figure 5d AMC-HN-8/si-circSHKBP1 + miR-766-5p inhibitor panel appears to be duplicated with part of a similar image in Figure 3B SCL-1/0 µM panel from Wang et al, 2020 (https://doi.org/10.2147/OTT.S251806).

When approached for an explanation, the authors provided their original data and answered our queries, but these do not sufficiently address the concerns.

As verifying the validity of published work is core to the integrity of the scholarly record, we are therefore retracting the article. The authors have been informed of this decision.

We have been informed in our decision-making by our policy on publishing ethics and integrity and COPE guidelines. 

The retracted article will remain online to maintain the scholarly record and will be digitally watermarked on each page as ‘Retracted’. 

